# Causal inference—so much more than statistics

**DOI:** 10.1093/ije/dyw328

**Published:** 2017-03-27

**Authors:** Neil Pearce, Debbie A Lawlor

**Affiliations:** 1 Department of Medical Statistics and Centre for Global NCDs, London School of Hygiene and Tropical Medicine, London, UK; 2 Centre for Public Health Research, Massey University, Wellington, New Zealand; 3 MRC Integrative Epidemiology Unit at the University of Bristol, Bristol, UK; 4 School of Social and Community Medicine, University of Bristol, Bristol, UK

## Introduction

It is perhaps not too great an exaggeration to say that Judea Pearl’s work has had a profound effect on the theory and practice of epidemiology. Pearl’s most striking contribution has been his marriage of the counterfactual and probabilistic approaches to causation.^1^ The resulting toolkit, particularly the use of counterfactual concepts and directed acyclic graphs (DAGs) has been extended by some epidemiologists to remarkable effect,^2^^,^^3^ so that some problems which were previously almost intractable can now be solved relatively easily. What we previously tried to understand using words, probabilities and numerical examples can now be explored using causal diagrams, so that mind-bending problems such as Berkson’s Bias can be explained and understood relatively easily.^4^^,^^5^

However, like *War and Peace* or *Finnegan’s Wake*, although most epidemiologists have by now heard of Pearl’s work, we suspect that relatively few have read it, at least not in the form of the original texts.^6^^,^^7^ It is therefore of considerable interest that Pearl, together with Madelyn Glymour and Nicholas Jewell, has now produced a primer *Causal Inference in Statistics.*^8^ Their motivation, set out in the preface, is that ‘statisticians are invariably motivated by causal questions’ but that the ‘peculiar nature of these questions is that they cannot be answered, or even articulated, in the traditional language of statistics’. They note that the development of new tools for causal inference over the decade has not excited statistical educators and that they are ‘essentially absent from statistics textbooks, especially at the introductory level’. We would add that the same is true in epidemiology, and that whereas there are debates about the relative prominence of these tools (as illustrated in recent papers and correspondence in the *IJE*^1^^,^^9–15^), it is essential that biostatisticians and epidemiologists alike are familiar and comfortable with these tools.

Given the complex nature of some of the concepts and methods covered, particularly for those who are not familiar with them, the book is remarkably accessible and clearly written. Chapter 1 introduces the fundamental concepts of causality, including the causal model. Chapter 2 explains how causal models are reflected in data, and how one might search for models that explain a given data set; graphical methods–in particular causal directed acyclic graphs (DAGs)–are introduced. Chapter 3 is concerned with how to make predictions using causal models. Chapter 4 then introduces the concept of counterfactuals, and discusses how we can compute them and what sorts of questions we can answer using them. The companion website [www.wiley.com/go/Pearl/Causality] is a valuable resource and provides answers to the many study questions throughout the book that help with learning and understanding (it is not straightforward to register with Wiley for this and you are initially taken to a site that appears to advertise the book only, but if you can negotiate the site, it will help you get the most out of the book).

## Key concepts

There are a number of key concepts and tools which are clarified in the book, but we will focus here on three: (i) the relationship between causality and statistics; (ii) concepts of causality; and (iii) causal DAGs.

### The relationship between causality and statistics–Simpson’s Paradox and the importance of context

The book starts with a simple example of Simpson’s Paradox showing how the results of a drug study in patients with an (unspecified) illness may look quite different depending on whether the findings are stratified by gender; if not, the drug appears to be decrease survival, whereas it actually increases survival within men and within women. This ‘confounding by gender’ can be readily addressed using stratification or any other form of adjustment, such as multiple regression. However, the same data are then re-presented with the name of one of the variables changed. The potential stratification variable is now high/low post-treatment blood pressure (BP), and it is known that the drug can lower blood pressure. The results are the same in the two examples (i.e. whether the strata are gender or post-treatment blood pressure, the drug decreases survival in aggregate data but improves it when stratified by gender or post treatment BP, with exactly the same magnitude and direction of results in both cases); only one variable name has been changed. But, in the former example, the correct result lies in the sex-stratified (segregated) results, whereas in the latter example it lies in the non-stratified by post-treatment blood pressure data (i.e. the aggregated results). Moreover, there is no statistical method which can help us to identify which of the two scenarios apply to a particular data set or analysis approach (aggregate or stratified). This can only be decided by information from outside the data set (e.g. that gender is a potential confounder and that the drug in part may increase survival by reducing BP).

Importantly, Pearl *et al.* use this example to illustrate the more general point that ‘causation is not merely an aspect of statistics; it is an addition to statistics, an enrichment that allows statistics to uncover workings of the world that traditional methods cannot’. Thus, we need to understand how and why causes influence their effects. This is not only essential in deciding how to analyse the data in a particular study in a particular population. It is also only by understanding how and why causes have their effects that we can also understand why causes may not have the same effects in other contexts. Thus, generalizability is a scientific process, not simply a matter of statistics (interestingly the book is titled *Causal Inference in Statistics*, thus implying that causal inference can involve statistics and vice versa, but they are not the same thing). This emphasis on the context in which causes occur (‘the causal story behind the data set’ as Pearl *et al.* refer to it), contrasts with much frequentist theory in which generalizability is mainly conceptualized in terms of sampling from larger (infinite) populations, and also much of randomized controlled trial (RCT) theory in which the focus is on effect estimation rather than aetiological understanding.

### Concepts of causality

Given current debates,^1^^,^^9–15^ it is also of considerable interest as to how causality is conceptualized by Pearl et al.:^8^

For our purposes, the definition of causation is simple, if a little metaphorical. A variable X is a cause of a variable Y if Y in any way relies on X for its value… X is a cause of Y if Y listens to X and decides its value in response to what it hears.

This is compatible with definitions that have been used in epidemiology for many years^16^ (see for example Lilienfeld,^17^ who stated that ‘a factor may be defined as a cause of a disease, if the incidence of the disease is diminished when exposure to this factor is likewise diminished’) as well as in some recent papers in the *IJE.*^1^^,^^12^

Note that there is no requirement here for any sort of intervention, or in fact any specification of how the value of X may change (or be changed). All that is required is that if the value of X were different, then the value of Y might also be different as a result.

It is particularly noteworthy that this inclusive pluralist concept of causation inherently involves causes which have been questioned in recent debates. In particular, causation is not restricted to specific actions (e.g. exercising 1 h/day), and ‘states’ such as ethnicity, gender, sex, obesity, hypertension and high cholesterol levels can also be causes. As with other causes of disease, some ‘states’ may be direct causes (e.g. the risk of breast cancer depends on the value of the variable ‘sex’), whereas others may only affect the risk of disease in certain contexts (e.g. in the context of sexism or racism). Furthermore, all of these different types of causes can be represented in DAGs, and we can attempt to estimate their causal effects (with varying degrees of success) while controlling for confounding and other sources of bias. Of course, one may wish to identify subgroups of causes with particular characteristics (e.g. states, actions), which are more or less prone to various types of bias. However, these represent differences between various types of causes; not between causes and ‘non-causes’.

### Directed acyclic graphs (DAGs)

DAGs are increasingly used in epidemiology, but in our experience they are not universally taught to epidemiologists. Even among early and mid-career epidemiologists, there appears to be a bimodal distribution of those who feel that all epidemiological research questions should be addressed using DAG(s) and those who seem to avoid them at all costs. We agree with others^9^ that DAGs are useful tools, but are neither necessary nor sufficient for causal inference. Nevertheless, they can be an extremely valuable way of illustrating the context (story) in which a causal question is being asked; in particular, they can illustrate the assumptions being made in causal analyses, and help us question their validity. For those less familiar with their use we provide a brief description of their key features in

Box 1. Our summary of the basics of directed acyclic graphs when used in causal inference

**Figure 1.**
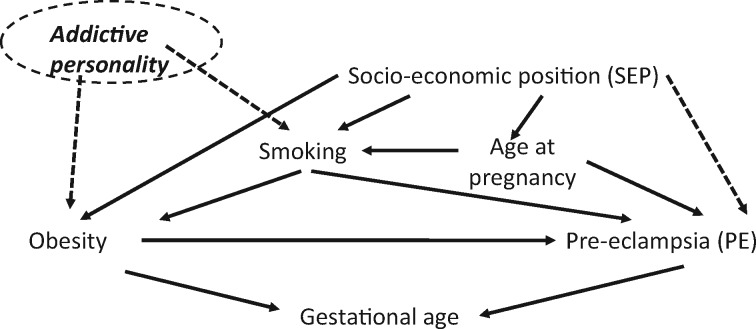
Illustrative example–directed acyclic graph for the hypothesis that obesity is causally related to pre-eclampsia

***Features of DAGs***:

1. Arrows (also known as ‘edges’ or ‘arcs’) connect ‘nodes’ which represent variables.
2. Arrows between nodes are directed. That is, only single-headed arrows can be included in a DAG.
3. Relationships are acyclic. That is, there are no series of arrows connecting nodes (i.e. no ‘paths’) that lead back to a node (variable) already in the path. The assumption is that a variable (in a given population at a given time) cannot cause itself.
4. Ideally, every variable that influences two or more other variables is shown in the DAG. In particular, the focus should be on those variables that influence the exposure and outcome. Though Pearl *et al.* in this book show situations where causal inference may be made without observing and adjusting for all potential confounders (e.g. where a confounding path can be blocked by conditioning on just one variable in the path) and even where none of the key confounders is observed [by using definite (known) causal mediators], these unobserved confounders need to be depicted in the graph (they are an essential part of the story/context).
5. Pearl *et al.*, like others, use the DAG concept of ‘back door path(s)’ to define confounding. A back door path is a series of arrows that link the exposure with the outcome; back door paths have an arrow going into the exposure at one end, and an arrow going into the outcome at the other end of the path. Some back door paths are shown in Figure 1. To remove confounding, we want to block all back door paths.

***The meaning of arrows and drawing DAGs:***

Arrows are drawn between any two variables according to the following criteria:

1. An arrow from one variable to a second indicates that you assume that it is plausible that the first variable causes the second.
2. Where there is no arrow between one variable and a second, this indicates that you assume that there is no causal relationship between the first and second variable.

***The absence of an arrow between two variables is very important:***

Indeed, if we think about confounding, the absence of an arrow is as important as the presence of one. For example, if we have an arrow from a variable to the outcome of interest, but no arrow (or path made up of a series of arrows) from that variable to the exposure, then we are assuming that the variable is not a confounder. If in reality the variable is related to the exposure, then any observed association between exposure and outcome might be biased as an estimate of causal effect due to residual confounding.

Box 1.

#### Using DAGs to decide what to adjust for and what not to adjust for–confounding and collider bias

Epidemiologists are very familiar with the concept of confounding; many lay people also understand this concept, as ‘to confound’ has a straightforward (non-technical) meaning (‘to fool’) which describes the problem of assuming causality in the presence of uncontrolled ‘confounders’. When DAGs are drawn appropriately they can clarify our assumptions about confounders, and can point to situations where observed and unobserved confounders can be controlled for. For example, when a confounding path (back door path) includes unobserved variables that do not influence exposure through any other path, the path may be blocked by controlling for observed confounders (Figure 1), assuming that these are accurately measured and appropriately adjusted for.

**Deciding what we should and should not adjust for on the basis of this DAG:**

**Scenario 1**

Assume that current knowledge does not imply a plausible effect of addictive personality on smoking or obesity, or that there is a direct relationship of SEP to PE risk, so these relationships (all shown with dashed arrows) are not included in the DAG in Scenario 1. We can make appropriate decisions about what needs to be adjusted for and what should not be adjusted for to obtain a valid estimate of the causal effect of obesity on PE if we assume that our DAG is correct [i.e. there are no other variables (nodes) or arrows that should be included] and that all variables are measured accurately (with little or no misclassification). We want to adjust for confounding–i.e. we want to block all back door paths. In this scenario there are four unblocked.

Unblocked backdoor paths

- PE-Age at pregnancy-SEP-Smoking-Obesity;
- PE-Age at pregnancy-SEP-Obesity;
- PE-Age at pregnancy-Smoking-Obesity;
- PE-Smoking-Obesity.

Because age at pregnancy is in the first three paths, we can block all three of those by adjusting for age at pregnancy only: assuming our DAG is correct and pregnancy age is accurately measured and so adjusting on it can fully block those paths. The last path does not include age; to block that we must control for smoking.

There is also one blocked path

- PE-Age at pregnancy -Smoking-SEP-Obesity; this is blocked because age and SEP collide on smoking.

However, we have said above that we have to adjust for smoking. When we do that, this path is unblocked and a spurious association between Pregnancy age and SEP is generated. In this scenario we are going to adjust for pregnancy age, which will block this path even when we adjust for smoking. To conclude, if we assume the DAG is correct and pregnancy age, obesity and PE are accurately measured (and there are no other sources of bias), then adjusting for pregnancy age and smoking will provide a valid causal estimate.

**Scenario 2**

New research/knowledge provides evidence that: (a) Addictive personality is relevant to our causal understanding of obesity on PE and must be added to the DAG as shown with dashed arrows (related to smoking and obesity) and (b) SEP is directly related to PE, also added to the DAG with a dashed arrow. This introduces one new unblocked path (in addition to the ones above):

- PE-SEP-Smoking-Addictive personality-Obesity.

We do not have a measure of Addictive personality or SEP, but we can block this path by adjusting for smoking (assuming our DAG is correct and no misclassification or other bias). We also still need to adjust for age and smoking to block the paths described above but now, when we adjust for smoking, we unblock the following blocked back door paths:

- PE-SEP-Addictive personality-Obesity;

Because we generate a spurious association between SEP and Addictive personality, if we do not have a measure of either of these in our dataset, the question is:

- Should we adjust for smoking to deal with confounding or should we not adjust for it because to do so would introduce collider bias?

The DAG cannot answer that–the answer lies in background knowledge and/or simulation studies that provide evidence for whether bias would be greatest with or without adjustment for smoking.

**Should we adjust for gestational age in either scenario?**

Very often in perinatal epidemiology gestational age is conditioned on–frequently this is done by excluding women who do not have a term delivery (i.e. where the baby is born before 37 weeks of completed gestation) either in the study design or analyses. In any analyses where exposure and outcome influence gestational age (as in this example, and commonly for many questions in this field), we should not do this. To do so potentially introduces a spurious association between Obesity and PE. In this specific case, that spurious association would be inverse and so this ‘collider’ bias could produce an effect estimate that is weaker than any true positive effect (biased towards the null). Note that whereas SEP would rarely be a plausible cause of ‘Age’ in this example, it is plausible to assume that SEP influences the age at which women start their family and hence become pregnant, with young women more likely to be from lower SEP and older from higher SEP.^19^

This primer also illustrates how conditioning (adjusting) on some variables–’colliders’–may introduce bias. Unlike the word ‘confounding’, ‘collider bias’ is not so intuitive and has no corresponding ‘lay’ meaning (it makes sense only with the use of DAGs). A collider is a node (representing a variable) that has two arrows coming into it on a path. Where a collider occurs, that back door path is blocked (Figure 1); there is thus no need to adjust for the collider as that path is already blocked. Importantly, adjusting on a collider opens up such a back door path, and thereby produces a spurious association between the two variables (e.g. exposure and disease) that it ‘connects’. Pearl *et al.* explain collider bias by using a theme that runs throughout the book, in which they define conditioning (or adjusting) as ‘filtering’ by the value(s) of the conditioning variable. In a very clear and simple way they point out that if Z is a collider for X and Y (i.e. the variable Z is influenced by X and Y; written in the book as Z = X + Y), and X and Y are independent of each other, and no other variables influence X, then conditioning on Z is the same as filtering on participants with the same value of Z. To take Pearl *et al.*’s simple additive example, if we know (only) that X = 3 for any participants that tells us nothing about the value of Y for those participants. But if we also condition (filter) on Z (as well as knowing that X = 3) within each stratum of Z, we now know the value of Y (if Z = 10, Y must = 7; if Z = 5, Y must = 2; if Z = 1, Y must = ‐2… and so on); by conditioning on (adjusting for) Z we have generated a spurious association between X and Y.

This fits with Simpson’s Paradox as illustrated in Chapter 1 of the book. Gender in the first example in Chapter 1 is a confounder and should be adjusted for, whereas post-treatment BP (the second example in Chapter 1) is a collider (influenced both by the drug and by recovery from the (unspecified) illness that the participants were suffering from) and should not be adjusted for.

In reality, few researchers would adjust for post-treatment BP in a study exploring the effect of a drug on an unspecified illness. Therefore, to illustrate collider bias further we use a more plausible example in Figure 1. This shows a DAG that might be drawn and used to inform what we should (and should not) adjust for to explore the causal effect of obesity on pre-eclampsia (PE) risk. The DAG shows our assumptions that: socioeconomic position (SEP) is at least plausibly causally related to obesity, smoking and age (at pregnancy), but not (directly) to pre-eclampsia, in scenario 1 that smoking is related to obesity and PE; that age is related to smoking, obesity and PE; and that both obesity and PE are related to gestational age at birth of the infant. These assumptions are based, to some extent, on research findings,^18–20^ but the DAG is also simplified for illustrative purposes and does not show all plausible influences on all variables represented in the DAG (see later discussion on limitation of DAGs). This DAG suggests that we can adjust solely for age at pregnancy and smoking to prevent confounding (including by SEP; see Figure 1). Thus, if we did not have a measure of SEP in our dataset, assuming that all other variables are accurately measured and the DAG is correct, we can obtain an estimate of the causal effect of obesity on PE risk. By contrast, we should not adjust for gestational age at birth as this is a collider on the path between PE and obesity (it is influenced by both of them since obese women are likely to have shorter duration pregnancies and those with PE are more likely to have their pregnancy induced or ended early by caesarean section). The importance of recognizing this is that many studies in perinatal epidemiology do restrict to term pregnancies only (either through excluding women who deliver preterm from being in the study or from being in analyses), without considering whether this might introduce bias.

##### Front door paths and the possibility of not having to measure confounders

**Figure 2.**
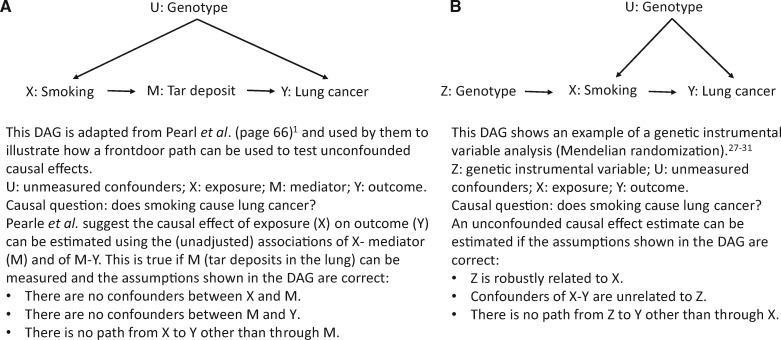
Pearl *et al.*’s front door and Mendelian randomization methods for testing unconfounded causal effects. A. Example of a front-door path to test unconfounded causal effects. B. Example of a Mendelian randomization approach to test unconfounded causal effects.

In section 3.4, Pearl et al. suggest that an unconfounded causal effect can be estimated using observational data, even when there are back door paths that cannot be blocked (because of unmeasured confounders). This is done using a front door path. A front door path is where there is one (or more) mediator(s) between the exposure and outcome and where there are no confounders of the exposure-mediator or mediator-outcome (Figure 2). The concept is that if there are unmeasured confounders between X (exposure) and Y (outcome) but no confounders between X and a mediator (M) or between M and Y, then the (unadjusted) associations of X and M and M and Y can provide the causal effect of X on Y. It feels like alchemy!

The example that Pearl *et al.* use to demonstrate this refers to an old argument that smoking does not cause lung cancer but rather that there are genes which influence both smoking and (independently) lung cancer risk, and thus confound the association of smoking with lung cancer. They present a thought experiment in which ‘tar deposits in the lung’ are a mediator between smoking and lung cancer, and show using a DAG (Figure 2), that an unconfounded causal effect can be estimated despite having no measure of the genetic confounder. If the DAG presented by Pearl *et al.* is correct, we agree that using this front door approach could provide a valid causal effect estimate. However, the example is fictional, and we struggle to imagine any situation in which there are not confounders between an exposure and a mediator, or mediator and outcome or misclassification of the mediator that is correlated with misclassification of the exposure.^21–23^ For us this front door approach is theoretically interesting but not likely to be widely applicable.

Mendelian randomization (MR), using genetic variants in genes that encode the nicotinic acetylcholine receptor as instrumental variables (IV), suggests a causal effect of greater intensity of smoking on lung cancer (Figure 2b).^24^^,^^25^ However, instrumental variable analyses (which Pearl *et al.* mention only in passing) have very different DAGs from that shown in Figure 2a, and a different set of assumptions (Figure 2b) from the more conventional approaches used in most of this book. These assumptions bring their own potential sources of bias. However, genetic variants are often valid IVs, and recent developments that provide valuable sensitivity analyses of the potential violation of the IV assumptions when using MR, mean that MR provides the potential for better causal inference in observational studies.^26–30^

#### Limitations of DAGs

We often find DAGs are useful for being explicit about assumptions of the causal context and helping researchers to better determine what should and should not be adjusted for. However, their limitations should also be considered.

Clearly, they can only ever be as good as the context (background information) that is used to draw them. For example, if they are drawn solely on the basis of available data rather than showing all key variables whether observed or unobserved, then causal effect estimates may be (residually) confounded. Perhaps more importantly, their use to guide analyses also depends strongly on the accuracy of the available data. This is true of all epidemiology, but may be particularly true when DAGs are used to imply that ‘causal’ analyses are straightforward and can determine complex causal paths, such as mediation with multivariable approaches applied to observational data.^21–23^

By their very nature DAGs assume that relationships are directed and acyclical. This will be true for many common biological and epidemiological processes, but there are also many exceptions in which truly cyclical or bidirectional relationships exist. It may be possible to resolve this with temporal knowledge. For example, if it is plausible that characteristic A at time one (A_t1_) influences characteristic B at a later time (B_t1+x_) which then goes on to influence characteristic A at a subsequent time [A_t1+y (where y is > x)_], and so on, these relationships can be represented in a DAG with no violation of its directed and acyclic properties. The DAG depicting these relationships treats characteristics at different time points as distinct nodes. However, causal processes cannot always be defined as directed and acyclical. This ‘linear’ approach to causality contrasts with complexity research involving non-linearity and feedback loops which cannot be readily summarized in a DAG.^31^

DAGs are also non-parametric, i.e. they make no assumptions about the nature or form of the causal relationships they depict, or even the direction (causative or preventive) of potential effects. Statistical interaction or effect modification can also be difficult to depict, although some methods have been proposed for doing this.^3^^,^^32^

Perhaps the largest limitation of DAGs is that they can be used to indicate possible sources of bias but cannot easily indicate how likely or how strong the biases may be. In one recent example relating to Berkson’s Bias,^4^^, ^^5^ DAGs were extremely powerful in helping to identify the nature of the bias, but not its strength. Berkson’s Bias produces extremely biased results when a study involves prevalent cases, a situation which cannot be easily represented by DAGs. If a study involves incident cases, the DAG remains the same, but (in this particular case) the bias becomes trivial.^4^ In our experience, creative colleagues can use DAGs to identify possible ‘collider bias’ in virtually any analysis, but this tells us little about whether the bias is likely to be large enough to be of practical importance.

Related to this, in some situations the distinction between what to adjust for and what not to adjust for is not simple even with a well-drawn DAG (Figure 1). For example, let us assume that following well-conducted research, it is clear that addictive personality is related to both smoking and obesity and therefore should be added to the DAG in Figure 1. Furthermore, new evidence suggests it is plausible that SEP influences preeclampsia risk through mechanisms that do not involve either maternal age at pregnancy or her smoking. This also needs adding to the DAG. However, we do not have data on either addictive personality or SEP; now our conclusions about what we should and should not adjust for are more complex. Above, before consideration of this new knowledge, we noted that we need only adjust for age at pregnancy and smoking. However, with the addition of this new knowledge, smoking is now a collider on the back door path PE-SEP-addictive personality-obesity and if we adjust for it we open that back door path (by generating a spurious association between addictive personality and SEP). (see Scenario 2; Figure 1). The question of whether the correct (or best) causal estimate is with or without adjustment for smoking cannot be answered from the DAG; though we would suggest that adjusting for it, given its proximal relationships to obesity and pre-eclampsia, is likely to be most important.^33^ In situations like this, the relatively new concept of collider bias can lead to a tendency to not adjust for a variable if there is a possibility of collider bias (‘collider anxiety’^4^), even if the collider bias is likely to be very weak whereas the uncontrolled confounding may be relatively strong. Greenland described this situation in a seminal paper in 2003.^33^ Although it will depend on the relative strengths of all associations between confounders and collider with exposure and outcome, in most situations more proximal confounding will be more important to control for. Greenland usefully provides suggestions for how one might undertake sensitivity analyses to test this, though they require appropriate contextual information to add value.^33^

These limitations highlight a general issue that the DAGs used throughout this book, as in the many methodological papers that advocate their use, are extremely simple (in order to illustrate specific methodological issues) and rarely reflect the reality of the numerous auxiliary hypotheses related to the main causal question (see below for more discussion). The DAG we show in Figure 1 is more complex than many in the primer, but it is a simple representation of the relationships that those of us working clinically and/or academically in this area know are relevant. A, by no means exhaustive, list of variables that ought also to be added to the DAG includes parity, change of partner, multiple pregnancy, placental function and fetal growth. For each of these we could go more ‘distal’, to add potential causes of the proximal common causes of exposure and outcome [i.e. distal ancestors of the main exposure (obesity) and outcome (PE)]. Where or when to stop is not clear. Software such as DAGitty and the suite of DAG functions in R (dagR) can deal with the most complex of DAGs and provide investigators with a minimum set of variables that should allow them to deal with potential confounding without resulting in collider bias. However, some studies using these packages fail to appropriately take account of theoretical context, but rather control for a large number of variables without clear reasoning and assume that this produces valid causal estimates from purely observational data.^34^

### Integrating diverse types of knowledge to answer causal questions

The use of methods such as triangulation, in which the aim is to integrate evidence from several approaches, that are chosen because they are sufficiently different to be likely to have different and unrelated key sources of bias that would be unlikely to produce the same result (due to these biases),^35^ may also be particularly important and even crucial, along with evidence from time trends and ecological studies. Going back to Pearl *et al.*’s front door example discussed above, evidence that smoking was a causal factor for lung cancer (rather than being confounded by genes or other factors) came several decades ago from such an integrative approach (including time trends in lung cancer incidence and mortality),^1^^,^^36^ rather than a theoretically correct but unrealistic DAG.

Thus, in epidemiology, the assessment of whether something is a cause is usually addressed through a process of integrating diverse types of knowledge, even if this is rarely acknowledged.^37^ Even when a particular study appears to be decisive, there are always assumptions, theories and contextual background information–from previous additional studies–that are necessary for a definitive judgement to be made.^38^ Thus, every process of causal identification and explanation involves evidence of a variety of types and from a variety of sources, and no single study is definitive. This is partly due to the Duhem/Quine’s thesis’ that a theory always relies on (but does not explicitly use) auxiliary hypotheses, and if some consequences of the theory turn out to be false, one of the auxiliary hypotheses rather than the theory may be incorrect.^39^ The fact that leaves may be observed to fly upwards in the wind does not necessarily refute the law of gravity but may instead refute auxiliary hypotheses (e.g. that there are no other forces operating that are stronger than gravity). Similarly, every epidemiological study involves the auxiliary hypothesis that no uncontrolled bias is occurring, and it may be this auxiliary hypothesis that is falsified rather than the main hypothesis of interest. As Pearl *et al.* point out in Chapter 3, even in a randomized controlled trial, a valid test of a theory (intervention) can only be obtained if a number of auxiliary conditions are met (full and/or unbiased participation, lack of misclassification, lack of contamination of the comparison group, etc.), and even a ‘perfect’ trial (which almost never exists) is intended (by design) to produce false-positive results 5% of the time (noting that most RCTs are designed to have sufficient power to detect a clinical/public health meaningful difference at the conventional 5% level of significance). Thus, interpretation of even the best possible trials always involves auxiliary information. These issues are considerably more acute in observational studies, but they are not unique to epidemiology. This is how most science works.^39^

Although any individual study can usually be represented in terms of counterfactual contrasts, which can in turn be represented in DAGs, it is difficult if not impossible to represent the overall process of epidemiological discovery and causal inference using these methods. Even if the available evidence is assessed at one particular point in time, the task of combining a wide variety of evidence from a wide variety of sources continues to be a matter of judgement,^10^ albeit one that can be aided by particular considerations such as those of Hill.^37^ None of this activity–the real ‘causal inference’–can be captured adequately in methods which focus on causal inference in a single study with a single DAG. Some of the commentaries in this issue suggest that DAGs do take account of all such relevant knowledge,^11^ but Krieger and Davey Smith challenge this.^34^

## Concluding remarks

Pearl *et al.* note in their preface that over the past decade the methods covered in this primer have resulted in a ‘transformative shift of focus in statistics research, accompanied by unprecedented excitement about the new problems and challenges…’. This has been accompanied by a number of excellent textbooks that develop Pearl’s work further (e.g. references 2 and 40). One of us (see references 1, 10 and 23) has been highly critical of the naive use of these methods and of the accompanying claims that they form a complete and sufficient theory of causal inference, rather than merely a useful set of tools which are appropriate in some situations but not others.^9^ However, we recognize the value and power of these methods when used appropriately and cautiously, together with other approaches such as triangulation.^35^ The problem is how to use these new methods critically and appropriately, rather than being captured by them in a manner which redefines and restricts what epidemiology is.^1^

This book thus represents a major resource for epidemiologists to learn the use of methods (e.g. structural causal models and DAGs) which have had major effects on the theory and practice of epidemiology in recent years. Our own experience in teaching is that these methods are extremely useful and would benefit from being introduced at an early stage of introductory epidemiology courses, provided that they are used ‘in context’ (i.e. studying the distribution and determinants of health in populations) rather than as a set of generic methods. They are not particularly difficult except to those who have been trained using different concepts and methods. If they are used (carefully and appropriately) from the beginning, then new students can grasp these concepts relatively easily–just as a teenager can usually use a modern cellphone easily whereas older generations may struggle. However, the limitations of these methods should also be considered in this teaching, and they should always be used as part of the epidemiological toolkit to address real-world problems (problem-based epidemiology^41–43^) rather than being used ‘out of context’ as a set of generic methods.
