# Erratum to: Causal inference—so much more than statistics

**DOI:** 10.1093/ije/dyz246

**Published:** 2019-12-03

**Authors:** Neil Pearce, Debbie A Lawlor

First published online: 27 March 2017, *Int J Epidemiol* 2016; 45: 1895–1903. doi: https://doi.org/10.1093/ije/dyw328

In the print version and online PDF of this article, in Scenario 2 in the extended caption to Figure 1 (page 1898), the first path should read ‘PE-Smoking-Addictive personality-Obesity’ rather than ‘PE-SEP-Smoking-Addictive personality-Obesity’ and in the following sentence ‘or SEP’ should not have been included.

In the online version of this paper, the extended caption was omitted from Figure 1 in both the downloadable Powerpoint slide and the html version that opens in a new tab. The html version also omitted the image and incorrectly included a section of text from the paper that did not pertain to Figure 1.

Further, the image was also omitted from the html version of Figure 2 that opens in a new tab, and that html version also incorrectly included a section of text from the paper that did not pertain to Figure 2.

The article has been corrected.

